# Gold Nanoparticle-Based Resuscitation of Cefoxitin against Clinical Pathogens: A Nano-Antibiotic Strategy to Overcome Resistance

**DOI:** 10.3390/nano12203643

**Published:** 2022-10-18

**Authors:** Ahmed Alafnan, Syed Mohd Danish Rizvi, Abdullah S. Alshammari, Syed Shah Mohammed Faiyaz, Amr Selim Abu Lila, Ahmed A. Katamesh, El-Sayed Khafagy, Hadil Faris Alotaibi, Abo Bakr F. Ahmed

**Affiliations:** 1 Department of Pharmacology and Toxicology, College of Pharmacy, University of Hail, Hail P.O. Box 2440, Saudi Arabia; 2 Department of Pharmaceutics, College of Pharmacy, University of Hail, Hail P.O. Box 2440, Saudi Arabia; 3 Department of Physics, College of Science, University of Hail, Hail P.O. Box 2440, Saudi Arabia; 4 Department of Physiology, College of Medicine, University of Hail, Hail P.O. Box 2440, Saudi Arabia; 5 Department of Pharmaceutics and Industrial Pharmacy, Faculty of Pharmacy, Zagazig University, Zagazig 44519, Egypt; 6 Central Administration for Drug Control, Egyptian Drug Authority “EDA”, Al Maadi 1347, Giza 11553, Egypt; 7 Department of Pharmaceutics, College of Pharmacy, Prince Sattam Bin Abdulaziz University, Al-kharj 11942, Saudi Arabia; 8 Department of Pharmaceutics and Industrial Pharmacy, Faculty of Pharmacy, Suez Canal University, Ismailia 41522, Egypt; 9 Department of Pharmaceutical Sciences, College of Pharmacy, Princess Nourah bint AbdulRahman University, Riyadh 11671, Saudi Arabia; 10 Department of Microbiology and Immunology, Faculty of Pharmacy, Minia University, Minia 61519, Egypt

**Keywords:** gold nanoparticles, drug delivery tool, Gram-negative pathogens, cefoxitin, nano-antibiotic

## Abstract

Gold nanoparticles have gained popularity as an effective drug delivery vehicle due to their unique features. In fact, antibiotics transported via gold nanoparticles have significantly enhanced their potency in the recent past. The present study used an approach to synthesize gold nanoparticles in one step with the help of cefoxitin antibiotic as a reducing and stabilizing agent. Cefoxitin is a second-generation cephalosporin that loses its potential due to modification in the porins (*ompK35* and *ompK36*) of Gram-negative pathogens. Thus, the present study has developed an idea to revive the potential of cefoxitin against clinical Gram-negative pathogens, i.e., *Escherichia coli* and *Klebsiella pneumoniae*, via applying gold nanoparticles as a delivery tool. Prior to antibacterial activity, characterization of cefoxitin–gold nanoparticles was performed via UV–visible spectrophotometry, dynamic light scattering, and electron microscopy. A characteristic UV–visible scan peak for gold nanoparticles was observed at 518 nm, ζ potential was estimated as −23.6 ± 1.6, and TEM estimated the size in the range of 2–12 nm. Moreover, cefoxitin loading efficiency on gold nanoparticles was calculated to be 71.92%. The antibacterial assay revealed that cefoxitin, after loading onto the gold nanoparticles, become potent against cefoxitin-resistant *E. coli* and *K. pneumoniae*, and their MIC_50_ values were estimated as 1.5 μg/mL and 2.5 μg/mL, respectively. Here, gold nanoparticles effectively deliver cefoxitin to the resistant pathogens, and convert it from unresponsive to a potent antibiotic. However, to obtain some convincing conclusions on the human relevance, their fate and toxicity need to be evaluated.

## 1. Introduction

Bacterial pathogens have developed different approaches to impede the effects of antibiotics in the past. Pathogenic strains have become capable of decreasing the permeability for antibiotics by modifying the outer membrane porins, developing pumps to efflux the antibiotics, and even directly destroying/modifying the antibiotics. Subsequently, the prevalence of antibiotic resistance has increased globally with time. Two of the most common Gram-negative sources of community or hospital-acquired infections, i.e., *Escherichia coli* and *Klebsiella pneumoniae* [1], have developed extended-spectrum beta-lactamase and modified outer-membrane porins in the recent past to escape the effect of the cephalosporin class of antibiotics [2,3,4,5,6,7]. The present study was focused on the resistance issues of *E. coli* and *K. pneumoniae* for cefoxitin, which is a second-generation cephalosporin. However, cefoxitin resistance in *E. coli* and *K. pneumoniae* has been associated with modification in non-specific outer membrane proteins *ompK35* and *ompK36* [6,7].

In the past, inorganic nanoparticles, specifically gold and silver nanoparticles, have allowed plausible solutions against resistance issues. They can form irreversible pores, disrupt the efflux pump, cause cell membrane disruption, and directly interact with bacterial biomolecules (nucleic acids, proteins, and lipids) [8,9,10,11]. In fact, several authors have used gold nanoparticles as a tool to deliver antibiotics to overcome resistance [12,13,14,15,16,17]. Gold nanoparticles are regarded as an optimal candidate for antibiotic delivery due to their biocompatibility and large ratio of surface area to volume. In addition, the attachment of the antibiotic to gold nanoparticles often tends to increase its potency by providing some additional bactericidal features. Increasing the cell wall porosity promotes the delivery of the attached antibiotic, and the direct interaction of gold nanoparticles with DNA could block the uncoiling and transcription processes. Interestingly, these features of gold nanoparticles will not only enhance the bactericidal activity but also assist in overcoming antibiotic resistance in some of the bacterial pathogens.

In 2017, cefotaxime resistance was successfully overcome by conjugating it on gold nanoparticles [17]. Similarly, ceftriaxone, ampicillin, and vancomycin resistance have been tackled efficaciously by applying gold nanoparticles as a delivery tool [15,18,19]. Thus, in the present study, gold nanoparticles loaded with cefoxitin were used to investigate their potential against cefoxitin-resistant pathogenic strains of *E. coli* and *K. pneumoniae*. Here, the cefoxitin-mediated synthesis of gold nanoparticles was performed, followed by characterization via dynamic light scattering, UV–visible spectroscopy, and electron microscopy. Further, the synthesized gold nanoparticles were tested against cefoxitin-resistant pathogenic strains of *E. coli* and *K. pneumoniae*. The implication of gold nanoparticles as a tool to deliver cefoxitin in resistant bacterial strains is one of the most feasible ways to tackle resistance issues. It is noteworthy to mention that authors have previously used a similar approach on third-generation cephalosporins successfully [14,15,17]. It is a universal fact that bacterial pathogens can find the means to become resistant with time to even newer classes of antibiotics by different mechanisms. In addition, scientists worldwide are expecting antibiotic resistance to become the next major issue after the COVID-19 pandemic. Thus, instead of spending time, intellectual efforts, and money on newer generations of antibiotics, if an older, ineffective antibiotic could be converted into a new and effective formulation, it will offer great relief for the scientific community.

## 2. Materials and Methods

### 2.1. Materials

Cefoxitin, solvents, media, and chemicals were purchased from Sigma Aldrich (St. Louis, MO, USA).

### 2.2. Synthesis of Gold Nanoparticles

Gold (III) chloride trihydrate salt solution (1 mM) in phosphate buffer (pH 7.2) was mixed with cefoxitin (250 μg) in a reaction mixture of 3 mL and incubated for 48 h at 40 °C [20]. After incubation, the reaction mixture color changed from yellow to ruby red, which confirmed the completion of the reaction and synthesis of gold nanoparticles. The synthesized cefoxitin-loaded gold nanoparticles were collected by centrifugating the mixture for 30 min at 30,000× *g*. Further, the nanoparticles were rinsed with 50% *v*/*v* ethanol and milli-Q water.

### 2.3. Characterization of Gold Nanoparticles

#### 2.3.1. UV–Visible Spectroscopy

To assess the conversion of gold salt solution to gold nanoparticles, a double-beam spectrophotometer (UV-1601, Shimadzu, Tokyo, Japan) was used. The range from 200 to 800 nm at 1 nm resolution was used to conduct the scan.

#### 2.3.2. Electron Microscopy

Transmission electron microscopy (TEM) for synthesized gold nanoparticles was performed on the Tecnai G2 Spirit TEM fitted with a BioTwin lens configuration (Hillsboro, OR, USA) after fixing the sample on a carbon-coated copper grid. The accelerating voltage of 80 kV was used to perform the analysis.

#### 2.3.3. Dynamic Light Scattering (DLS)

The DLS approach was applied to estimate the hydrodynamic diameter and zeta potential of the synthesized nanoparticles by using the Malvern Zetasizer Nano-ZS (ZEN3600, Malvern Instrument Ltd., Malvern, UK). Prior to analysis, the synthesized gold nanoparticles were sonicated for 60 s and filtered by membrane filters (0.45 μm). The mean size of nanoparticles was estimated by taking a sample in a 1.5 mL disposable (DTS0112) cuvette. However, the particle surface charge (zeta potential) was measured by using DTS1070 disposable cuvettes [21].

### 2.4. Loading Efficiency Estimation

Cefoxitin loading efficacy on gold nanoparticles was estimated by using the methodology of Gomes et al. [22]. The reaction mixture of synthesized gold nanoparticles was centrifuged at 30,000× *g* for 30 min, and the supernatant was collected carefully. Free cefoxitin (unbounded cefoxitin) in the supernatant was calculated spectrophotometrically at 235 nm [23] by using the cefoxitin calibration curve pre-constructed at different concentrations. The following equation [22] was applied to calculate the loading efficacy percentage:

Loading efficacy (%) = [(X − Y)/X] × 100 (1)

X is the total amount of cefoxitin added during gold nanoparticle synthesis, whereas Y is the amount of free cefoxitin present in the supernatant.

### 2.5. Antibacterial Assay

#### 2.5.1. Bacterial Strains

*Escherichia coli* (Seq# 427812372404) and *Klebsiella pneumoniae* (Seq# 427811998026) strains were collected from Hail General Hospital, Hail, to investigate the antibacterial activity of cefoxitin-loaded gold nanoparticles. Each bacterium was freshly inoculated in nutrient broth and incubated overnight at 37 °C. The turbidity was achieved to 0.5 McFarland standard (1.5 × 108 CFU/mL) with nutrient broth before evaluating the nanoparticles for antibacterial activity.

#### 2.5.2. Agar Well Diffusion

The agar well diffusion technique [24] was applied to determine the efficacy of pure cefoxitin and cefoxitin-loaded gold nanoparticles. First, a 100 μL inoculum of each bacterial strain was swabbed twice on the surface of Mueller–Hinton agar plates to form a uniform lawn. Four holes (two 4 mm and two 8 mm diameter) were punched aseptically with a cork borer, and 50 μL (3 μg/well) and 100 μL (6 μg/well) of pure cefoxitin and cefoxitin-loaded gold nanoparticles were added to the wells. Petri plates were incubated at 37 °C overnight, and inspected for the zone of inhibition in mm. The experiment was repeated in triplicate, and the inhibition zone was measured as mean ± standard deviation.

#### 2.5.3. Minimal Inhibitory Concentration (MIC)

The microbroth dilution method [25] was applied to calculate the MIC of cefoxitin-loaded gold nanoparticles against the tested strains of *Escherichia coli* and *Klebsiella pneumoniae*. The concentration range of 0.56 to 36 μg/mL for cefoxitin (loaded onto gold nanoparticles) was adjusted with sterile nutrient broth in a 96-well microtiter plate. The same concentration range was used for cefoxitin alone for comparative analysis. In each well, 10 μL of tested bacterial strain at 1.5 × 10^8^ CFU/mL was inoculated. Plates were incubated overnight at 37 °C after inoculation, and an ELISA reader was used to measure the viability at 625 nm. The minimum concentration that inhibited the growth of the tested strains was recorded as the MIC, and phosphate buffer was used as a control. Three independent experiments were set up to obtain the MIC result as mean ± standard deviation.

## 3. Results and Discussion

### 3.1. Cefoxitin-Loaded Gold Nanoparticle Synthesis

**Figure 1:**
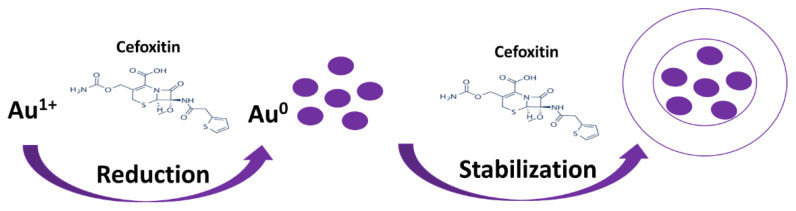
Scheme for the synthesis and stabilization of gold nanoparticles by cefoxitin.

There are multiple ways to synthesize gold nanoparticles of the desired size and property [26,27,28]. They include the use of chemical reducing agents such as sodium borohydrate, citrate, etc. However, post-synthesis, a stabilizing/capping agent is necessary to stabilize the synthesized gold nanoparticles [29,30]. Researchers have developed an idea to use natural reducing agents such as bromelain, trypsin, and herbal extracts, which could act as both reducing and capping agents to make the process easier, and this is termed the one-pot synthesis method [20,31,32]. However, the loading or attachment of drugs to the stabilized gold nanoparticles is a tedious task that includes the use of chemical cross-linking agents such as EDC (1-ethyl-3-(3-dimethylaminopropyl) carbodiimide) [17,33,34]. Thus, nano-scientists have developed protocols where the drug itself acts as a reducing as well as capping agent to minimize the use of chemicals. Similarly, in the present study, cefoxitin was used to mediate the synthesis of gold nanoparticles by acting as a reducing and capping agent (Figure 1). This strategy provided us with a method to synthesize stable nanoparticles and achieve subsequent cefoxitin attachment in one step. Earlier reports on β-lactam antibiotics suggested that their amine group participates in the reduction of gold and silver salts, and no changes in the β-lactam ring were observed even after the capping/stabilization of nanoparticles [16,35]. In fact, one-pot synthesis via antibiotics (without the use of chemical reducing/stabilizing/cross-linking agents) will minimize the probability of any false antibacterial results because of residual chemicals used in the processing of nanoparticles. Several authors have applied an antibiotic-mediated gold nanoparticle synthesis strategy in the recent past [12,14,15,16]. Interestingly, they have found enhanced potential against pathogenic bacterial strains.

In the current study, cefoxitin (250 μg) was added to a 3 mL reaction mixture containing 1 mM of gold salt in phosphate buffer (pH 7.2). It has been observed that the pH influences the zeta potential of the gold nanoparticle monolayer [36]. However, the pH of 7.2 for the present study was selected on the basis of an earlier reported method [20]. Further, the reaction mixture was kept at 40 °C for two days to complete the formation of gold nanoparticles after successful reduction and capping. Time-to-time physical observation and UV–vis scanning were performed to check the success of the synthesis protocol. The color of the reaction mixture changed from yellow to the characteristic ruby red color, which confirmed the formation of gold nanoparticles. The sample was further characterized by UV–vis scanning, zeta sizing and potential measurement, and electron microscopy.

### 3.2. Characterization of Cefoxitin-Loaded Gold Nanoparticles

**Figure 2:**
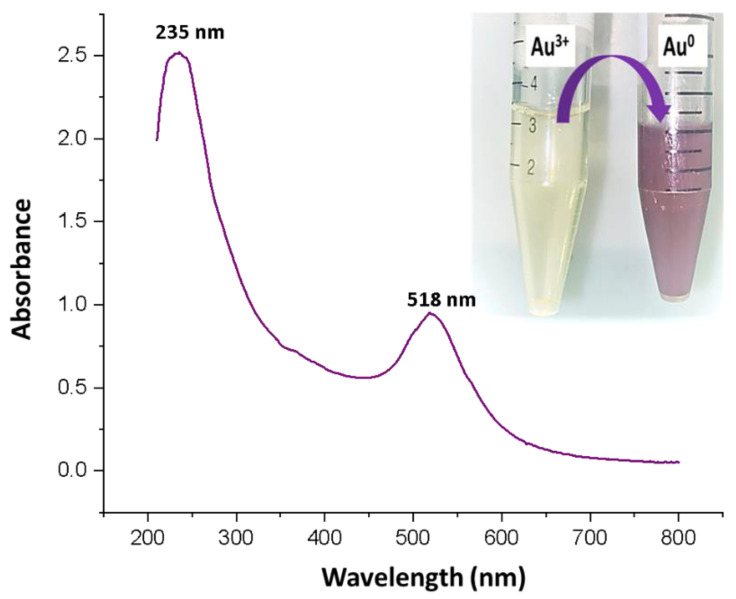
UV–vis spectroscopy scan of the cefoxitin-synthesized gold nanoparticles. (Inset) Physical observation of the change in color from yellow to ruby red.

Gold nanoparticles show characteristic surface plasma resonance (SPR) in the visible range of 500 to 600 nm. In the current study, the SPR band peak for the synthesized gold nanoparticles was observed at 518 nm (Figure 2). In addition, a peak at 235 nm was also observed that corresponded to the cefoxitin loaded onto gold nanoparticles [23]. However, the color change from yellow to ruby red, shown in Figure 2 (inset), also corresponds to the successful synthesis of gold nanoparticles. Thus, cefoxitin successfully mediated the synthesis of stable gold nanoparticles. A similar approach was used for the synthesis of cefotaxime, ceftriaxone, delafloxacin, and vancomycin–gold nanoparticles in the recent past [12,14,15]. Interestingly, dual peaks were observed for cefotaxime (260 nm), ceftriaxone (241 nm), delafloxacin (290 nm), and vancomycin (278 nm), and their synthesized gold nanoparticles, at 532 nm, 536 nm, 530 nm, and 524 nm, respectively [12,14,15]. In fact, if the antibiotics were attached to the gold nanoparticles by using EDC, two peaks—one for the antibiotic and one for the gold nanoparticles—were reported [17,34]. Hence, it can be safely stated that cefoxitin was successfully loaded onto gold nanoparticles after synthesis in the current study.

**Figure 3:**
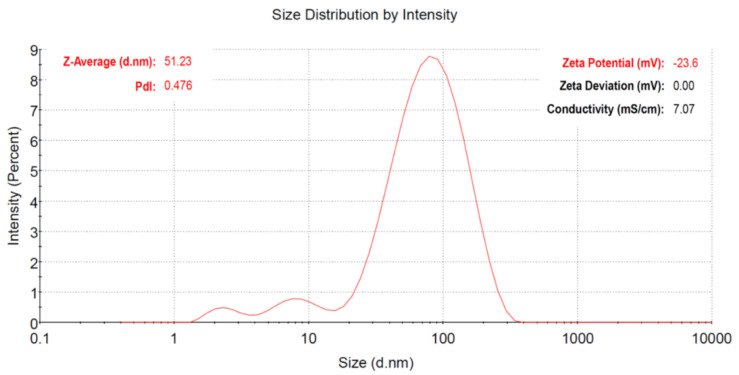
Size distribution intensity plot generated by dynamic light scattering for cefoxitin-synthesized gold nanoparticles. (Inset) Zeta potential of the synthesized gold nanoparticles.

To determine the size distribution of the cefoxitin-synthesized gold nanoparticles in the dispersion, the dynamic light scattering (DLS) technique was used. In fact, during DLS analysis, the arbitrary variations in the light intensity scattered by the dispersion of nanoparticles are used to estimate the size [37]. The Z-average mean size was estimated as 51 nm for the cefoxitin-synthesized gold nanoparticles in the present study (Figure 3). On the other hand, zeta potential was used to assess the surface charge on gold nanoparticles, which showed their colloidal dispersion stability [38]. It was actually dependent on the level of electrostatic repulsion within the similarly charged particles in the dispersion. In the current study, the zeta potential value of cefoxitin-synthesized gold nanoparticles was calculated as −23.6 mV (Figure 3 inset). It has been reported that particles with higher +ve or −ve zeta potential (>±20 mV) repel each other, and will not be aggregated easily [39]. Hence, the cefoxitin-synthesized gold nanoparticles of the present study were found to be stable. However, the high negative charge might be attributed to the presence of functional groups in the cefoxitin antibiotic loaded on the surfaces of the gold nanoparticles.

**Figure 4:**
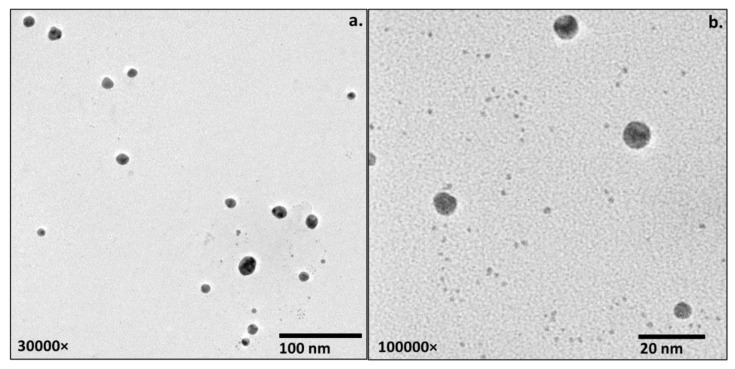
TEM images of cefoxitin–gold nanoparticles (**a**) at 30,000× and (**b**) at 1,000,000×.

Transmission electron microscopy (TEM) was used to depict the size of the inorganic core of the cefoxitin–gold nanoparticles. Two different magnifications were used for TEM imaging: 30,000× (Figure 4a) and 1,000,000× (Figure 4b). TEM images for cefoxitin–gold nanoparticles revealed that the cefoxitin–gold nanoparticles were poly-dispersed, with a size of 2–12 nm. No aggregation was observed in the TEM images, which corresponds to effective capping/stabilization via cefoxitin. Size differences in the cefoxitin–gold nanoparticles evaluated by DLS (51 nm) and TEM (2–12 nm) could be observed in this study that might be due to the different principles of size calculation. TEM sizing is based on transmission electrons and provides information of the inorganic core, whereas DLS estimates the size of nanoparticles in a hydration state, which includes the information of the solvent layer adhered to them in the liquid medium [40,41,42,43,44]. Thus, the size estimation by DLS is relatively higher than that by TEM. This aspect of the size measurement of gold nanoparticles has been reported in several previous investigations as well [12,13,14,15].

### 3.3. Loading Efficacy

One crucial parameter for the characterization of nanoparticles and their subsequent application is the calculation of the loading efficiency of the drug onto nanoparticles. In this study, cefoxitin’s loading efficiency onto gold nanoparticles was estimated as 71.92%. Here, 250 μg of cefoxitin was initially added to the reaction mixture; however, 179.8 μg (approx. 180 μg) was estimated to be loaded onto the gold nanoparticles. This indicates no substantial loss of cefoxitin during the processing conditions, and the efficacious loading of cefoxitin on the surfaces of the gold nanoparticles.

### 3.4. Antibacterial Assay

**Figure 5:**
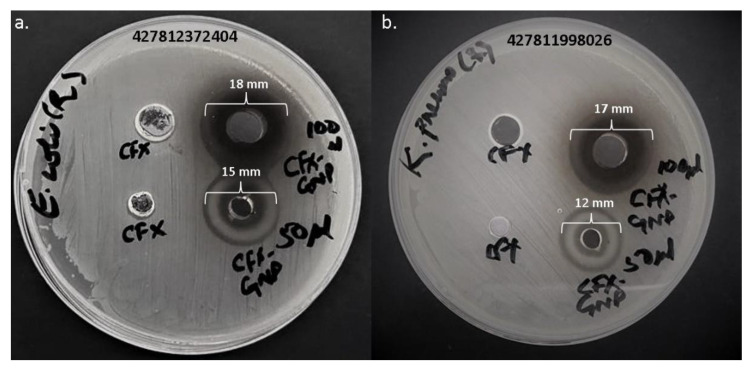
Antibacterial activity of cefoxitin (CFX) and cefoxitin–gold nanoparticles (CFX-GNP) against (**a**) *Escherichia coli* and (**b**) *Klebsiella pneumoniae*.

The antibacterial activity of cefoxitin–gold nanoparticles and pure cefoxitin was estimated against clinical strains of *Escherichia coli* and *Klebsiella pneumoniae*. The well diffusion technique was applied to preliminarily screen the potential of pure cefoxitin and cefoxitin–gold nanoparticles (Table 1, Figure 5). At a 3 μg/well concentration, cefoxitin–gold nanoparticles showed 12 mm and 15 mm zones of inhibition against *K. pneumoniae* and *E. coli*, respectively. At a 6 μg/well concentration, the zone of inhibition increased to 17 mm and 18 mm against *K. pneumoniae* and *E. coli*, respectively. However, at both the concentrations, 3 and 6 μg/well, pure cefoxitin showed no antibacterial activity against the tested strains. Thus, it can be suggested that the gold nanoparticles significantly revived cefoxitin’s potential against the tested bacterial strains.

**Figure 6:**
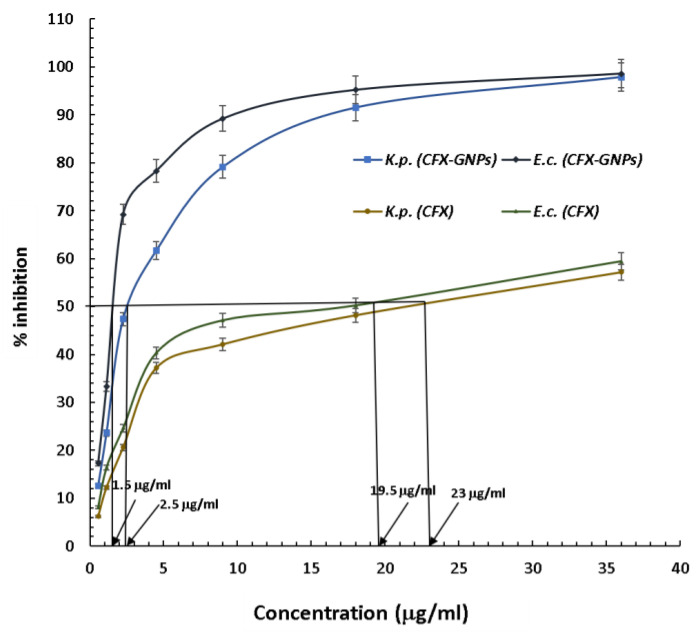
MIC_50_ of cefoxitin–gold nanoparticles and pure cefoxitin against *Escherichia coli* and *Klebsiella pneumoniae*.

It is noteworthy to mention that cefoxitin (a second-generation cephalosporin) was once considered as an alternative if the bacterial strain developed resistance towards other second-generation cephalosporins, due to its resistance towards β-lactamase/cephalosporinase [45]. However, past reports suggested that bacterial strains have gained resistance towards cefoxitin as well by alternate mechanisms [6,7,46,47,48]. It has been reported that cefoxitin resistance in *K. pneumoniae* and *E. coli* is linked with porins or non-specific outer-membrane proteins *ompK35* and *ompK36* [6,7]. In fact, porins are water-filled channels that allow the diffusion of the β-lactam antibiotic via the outer membrane. However, cefoxitin (β-lactam antibiotic) resistance is due to the loss or modification of these porin channels [6]. Gold nanoparticles have the capability to form pores in the outer membranes of Gram-negative bacterial strains and cause cell membrane disruption [8,9,10]. Hence, they can deliver the antibiotic attached to them in a more efficient manner, even though the porins are lost or modified. The current study showed that cefoxitin resistance was overcome after loading onto gold nanoparticles. These results are supported by other studies conducted on third-generation cephalosporins (cefotaxime and ceftriaxone) in the recent past [14,15]. They used different Gram-positive and Gram-negative strains of non-clinical origin to test the antibacterial efficacy [14,15]. However, the current study used a second-generation cephalosporin against the clinical uro-pathogenic resistant strains of *K. pneumoniae* and *E. coli*. An additional benefit of gold nanoparticles as an antibiotic carrier is that they offer a large surface area to deliver a good amount of antibiotic to the bacterial cell. Therefore, it is anticipated that the gold nanoparticles might have delivered a larger cefoxitin quantity to the tested strains in comparison to cefoxitin alone. The gold nanoparticle itself is capable of interacting with the biomolecules of bacterial cells, and causes substantial damage to the cell. Thus, cefoxitin’s successful delivery might have provided a synergistic attack on the bacterial cell. Further, the minimum inhibitory concentration (MIC_50_) was calculated for cefoxitin–gold nanoparticles against *K. pneumoniae* and *E. coli*. The MIC_50_ for cefoxitin–gold nanoparticles was estimated as 1.5 μg/mL against *E. coli* and 2.5 μg/mL against *K. pneumoniae* (Figure 6). On the other hand, cefoxitin alone showed an MIC_50_ of 19.5 μg/mL and 23 μg/mL against *E. coli* and *K. pneumoniae*, respectively. All these results evidently prove that nanotechnology could augment the potency of cefoxitin via loading onto gold nanoparticles. Importantly, an easy one-pot synthesis approach was applied in the present study (without the use of any external chemical reducing/capping agent) to produce effective cefoxitin–gold nanoparticles. Conclusively, it can be proposed that gold nanoparticles, as a cefoxitin carrier, might be applied in the future to overcome/reduce the probability of cefoxitin resistance.

## 4. Conclusions

The conversion of an ineffective antibiotic into a potent antibiotic nano-formulation is an appealing idea in the current scenario of increasing resistance towards different classes of antibiotics. The present study used a one-pot gold nanoparticle synthesis approach to revive cefoxitin’s potential against resistant clinical pathogens. Here, cefoxitin acted as a reducing and capping agent to produce gold nanoparticles of high stability (ζ potential −23.6 ± 1.6), with 180 μg of cefoxitin (loading efficacy of 71.92%) loaded onto them. The antibacterial assay clearly indicated that cefoxitin, after loading onto gold nanoparticles, became significantly more potent against the tested clinical strains of *Escherichia coli* and *Klebsiella pneumoniae*. Meanwhile, at the same concentration, pure cefoxitin showed no activity against both strains. The results of the present study suggested that loading an antibiotic (cefoxitin) onto gold nanoparticles could provide a good strategy to resolve the resistance-related issues. However, in vivo studies are warranted to investigate the toxicity and fate of cefoxitin–gold nanoparticles before considering their applicability.

## Figures and Tables

**Table 1:** Antibacterial activity assessment by well diffusion method.

Concentration	Zone of Inhibition	
	*Escherichia coli*	*Klebsiella pneumoniae*
3 μg/well (Cefoxitin alone)	-	-
3 μg/well (Cefoxitin–GNPs)	15 mm	12 mm
6 μg/well (Cefoxitin alone)	-	-
6 μg/well (Cefoxitin–GNPs)	18 mm	17 mm

## Data Availability

Not applicable.
